# Quantification of Dendritic Spines Remodeling under Physiological Stimuli and in Pathological Conditions

**DOI:** 10.3390/ijms22084053

**Published:** 2021-04-14

**Authors:** Ewa Bączyńska, Katarzyna Karolina Pels, Subhadip Basu, Jakub Włodarczyk, Błażej Ruszczycki

**Affiliations:** 1 Nencki Institute of Experimental Biology, Polish Academy of Sciences, 3 Pasteur Street, 02-093 Warsaw, Poland; e.baczynska@nencki.edu.pl (E.B.); k.pels@nencki.edu.pl (K.K.P.); j.wlodarczyk@nencki.edu.pl (J.W.); 2 Department of Computer Science and Engineering, Jadvapur University, Kolkata 700032, India; subhadip@cse.jdvu.ac.in

**Keywords:** dendritic spines, dendritic spine analysis, spine remodeling, synaptic plasticity, structural plasticity, neuronal remodeling, dendritic spine morphology

## Abstract

Numerous brain diseases are associated with abnormalities in morphology and density of dendritic spines, small membranous protrusions whose structural geometry correlates with the strength of synaptic connections. Thus, the quantitative analysis of dendritic spines remodeling in microscopic images is one of the key elements towards understanding mechanisms of structural neuronal plasticity and bases of brain pathology. In the following article, we review experimental approaches designed to assess quantitative features of dendritic spines under physiological stimuli and in pathological conditions. We compare various methodological pipelines of biological models, sample preparation, data analysis, image acquisition, sample size, and statistical analysis. The methodology and results of relevant experiments are systematically summarized in a tabular form. In particular, we focus on quantitative data regarding the number of animals, cells, dendritic spines, types of studied parameters, size of observed changes, and their statistical significance.

## 1. Introduction

Cognitive processes, as well as most brain diseases, involve functional modification of neuronal networks through reorganization of existing synapses [1,2,3]. Synapses undergo dynamic changes upon environmental stimuli, and therefore they are believed to play a major role in brain plasticity [4,5,6]. Most neuroreceptors of the excitatory synapses are located on small membranous protrusions, called the dendritic spines, whose structural geometry correlates with the strength of synaptic connections [7,8]. The synaptic plasticity is therefore inherently related to remodeling of dendritic spines. This remodeling, when observed in microscopic images, predominantly manifests itself in the form of morphological changes of dendritic spines [9,10], or the effect on spine density due to the formation or elimination of dendritic spines [11]. For example, the newly formed spines were observed to be thin, and upon stimulation they started to maturate, creating more stable synapses [12]. Numerous neuropsychiatric and neurodegenerative disorders, such as major depressive disorder, schizophrenia, Fragile X syndrome, or Alzheimer’s disease, reveal abnormalities in morphology and density of dendritic spines, indicating obstructions in the transformation of immature spines into mature ones [13,14]. Therefore, an understanding of the mechanisms underlying dendritic spine reorganization, in a context-dependent manner, is extremely important, and may lead to the development of a new strategy in the treatment of brain diseases targeting molecular pathways associated with the regulation of spine structure.

**Figure 1:**
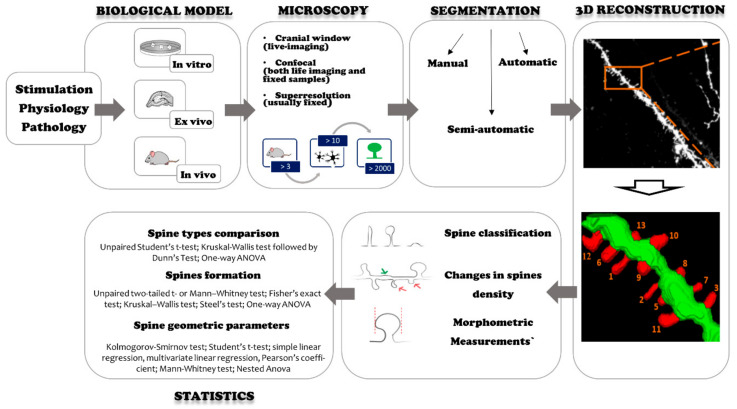
Typical experimental workflow. Green color indicates the dendrite, red color indicates the dendritic spines.

In this article, we review various experimental approaches to assess quantitative features of dendritic spines under physiological and pathological conditions. We compare different methodologies (Figure 1), focusing on testing conditions, sample preparation, data analysis, image acquisition, sample size, and statistical analysis. The methodologies are summarized in Table 1 for experiments where underlying physiology is changed or stimulation is performed, and in Table 2 for experiments related to brain diseases. We also extracted quantitative data regarding the number of animals, cells, dendritic spines, types of studied parameters, the size of observed changes, and their statistical significance.

## 2. Functional Meaning of Spine Remodeling

Most of the excitatory synapses in the mammalian brain occur mainly on dendritic spines located on dendrites [10]. In contrast, inhibitory synapses are formed on the cell bodies, shafts of dendrites, and axonal initial segments. The postsynaptic part of excitatory synapses differs from inhibitory synapses, not only in the type of neurotransmitter receptors, but also in their morphology and molecular organization. Due to the distinctive structure of dendritic spines, much more is known about the excitatory than inhibitory synapses [55,56].

**Figure 2:**
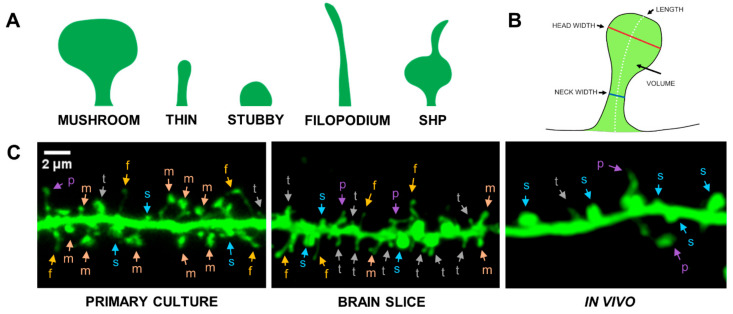
Morphological diversity of dendritic spines. (**A**) Spine shape classification: mushroom (m), thin (t), stubby (s), filopodium (f), SHP-spine head protrusion (p); (**B**) definition of morphometric parameters; (**C**) microscopic images of dendrites covered with dendritic spines obtained from in vitro (primary culture), ex vivo (brain slice), and in vivo (cranial window) imaging. Scale bar: 2 μm.

Dendritic spines, as a dynamic structure, can change their shape spontaneously or as a response to physiological or pathological stimulation. Dendritic spines also undergo dynamic turn-over, i.e., spine elimination and de novo formation [1,3,10,57,58]. These processes of changing the shape and/or number of dendritic spines are called structural synaptic plasticity and occur continuously, with such changes persisting in the brains of adult individuals [59]. The dendritic spine shape correlates with synapse strength and function, and various groups of dendritic spines shape can be distinguishable, e.g., mushroom-shaped, thin, stubby, filopodial, spine-head protrusion (SHP), see Figure 2, and sometimes cup-shaped spines. In the adult brain, about 65% of spines represent mushroom-shaped, 20% thin, while 15% are the remaining spine shape groups [60,61,62,63,64]. It is assumed that the mushroom spines are mature, therefore defined as memory spines, while thin spines are immature and called learning spines [1]. Mushroom spines with a thin neck and a large head form postsynaptic density (PSD)—containing ion channels, neurotransmitter receptors, cell adhesion molecules (CAM), scaffolding proteins and other intracellular signaling proteins [55,56,61]. The volume of the dendritic spine head correlates with the accumulation of postsynaptic glutamatergic AMPA receptors [7,65]. Evidence for the stability of this group of spines was provided by in vivo structural plasticity studies showing that mushroom-shaped dendritic spines can be stable over months or even years [27,63,66,67]. In addition, research on associative learning shows that mushroom spines can also have two types (excitatory and inhibitory) of synapses simultaneously. These double synapses may occur de novo or by adding inhibitory synapses to the already existing dendritic spine with a single excitatory synapse [68]. The stubby spines do not have distinguishable head and neck [69]. However, there are indications that stubby spines may in fact be mushroom-shaped spines with a very short neck [70]. Thin spines are smaller than mushroom spines, have a faintly distinct head and a thin neck, and form functional synapses less frequently than mushroom spines. The consequence of the unstable structure of these spines is their greater potential for plastic changes [27,67,71]. In contrast, filopodial spines are the smallest membranous protrusion. This group of spines, similar to the thin spines, is believed to constitute immature forms of dendritic spines that are precursors of mature mushroom spines [60,72,73,74]. Spine-head protrusions (SHPs) are a filopodial protrusion that occurs mainly on mature mushroom-shaped spines being a transient form of structural reorganization of the spine in response to stimulation [22,75]. It should be emphasized that despite the existence of an arbitrarily defined classification of dendritic spine shapes and hence their function, the spines presumably exhibit shape continuum [76].

The formation of new spines, and the morphological diversity, both depend on neuronal excitability during physiological processes such as learning and memory or pathological processes underlying brain pathologies. Studies on the influence of long-term potentiation (LTP) on the structure of synapses, show that dendritic spines undergo plastic changes upon stimulation. Experiments using two-photon microscopy, have shown that induction of LTP in hippocampal slices causes de novo formation of dendritic spines [77]. Numerous studies indicate that in response to LTP, the existing dendritic spines are also structurally altered. The basis for morphological changes of dendritic spines is the reorganization of the actin cytoskeleton. It was observed that an increase in the heads of spines, as well as widening and shortening of their necks, occurred 2 min after LTP induction and persisted for several hours [78,79]. Moreover, studies on anesthetized rats showed a significant increase in the volume of thin and mushroom-shaped dendritic spines during LTP [80]. In vitro studies on neuronal cultures have also shown that LTP increases the size of the dendritic spine head and stabilizes the newly formed spines [81,82,83,84]. Additionally, enlargement of the dendritic spine heads during LTP is associated with the incorporation of AMPA receptors into the postsynaptic membrane [85,86,87,88]. These studies show that an increase in the volume of dendritic spines affects the efficiency of the neuronal network, causing changes in synaptic transmission.

Thus, dendritic spines comprise fundamental computational units associated with synaptic plasticity and behavior [3,89,90,91]. Therefore, for decades, the mechanisms underlying dendritic spine remodeling, under physiological changes, stimulation or in pathological conditions, have been studied in correlation with electrophysiology and behavior.

## 3. Experimental Methodology

### 3.1. Physiological Conditions, Stimulation and Disease Models.

The processes of dendritic spines remodeling can be observed on different levels of biological complexity, in different testing conditions—in vitro, ex vivo, and in vivo, and within different species, however, contemporary neuroscience is mainly focused on animal models combined with translational human research through post mortem or biopsies analysis [92,93]. Diversity of methodological approach in animal models (physiological, pharmacological, and genetic models) have enhanced our understanding of the molecular basis of brain plasticity. Physiologically induced changes in animal behavior consist of exposure to specific environmental stimuli, but in pharmacologically induced models those changes are induced by acute or chronic drug treatment. Moreover, genomic technologies generate genetically modified animals (knock-out, knock-in, and conditional mutant mice) creating a powerful tool to study mechanisms underlying both physiological [18,19,20] as well as pathological processes. Furthermore, it is commonly practiced to introduce a viral vector plasmid into a specific brain region that locally regulates gene expression in adult animals, opening enormous possibilities to study brain plasticity at structural and functional level. Recently developed technology harnessing human iPSC–derived cortical neurons transplanted in the adult mouse cortex reveals a new direction of research in which in vivo imaging of cortical, human-originating, dendritic spines becomes possible [42].

To highlight the role of dendritic spines in various context-dependent conditions, we have summarized dendritic spine remodeling in physiological processes and upon stimulation (Table 1) induced by postsynaptic receptor antagonism [15], postsynaptic receptor agonism [16,17], genetic modifications [18,19,20], Chemically induced LTP [21,22,23,24,25], LTD (long-term depression) [26], sensory experience [27,28], spatial memory and learning [29], physical environmental stimuli [30,31,32]. Pathological processes (Table 2) were categorized into brain diseases underling neurodegenerative (Alzheimer’s disease [33,34,35], Parkinson disease [36], Fragile X Syndrome [37,38], Down Syndrome [39], Rett Syndrome [40,41], Autism Spectrum Disorder [42], Huntingtin Disease [43]), neuropsychiatric (Schizophrenia [44], Depression [45,46,47]), Stroke [48], Epilepsy [49], and infectious diseases such as Prion Disease [50], HIV infection [51], Influenza Infection [52], Toxoplasmosis [53] and Antiviral Responses [54].

### 3.2. Dendritic Spines Labelling and Sample Preparation

Dendritic spine imaging can be performed on primary neuronal culture, organotypic neuronal cultures, acute brain slices, in adult animals during live cell imaging or after chemical cell fixation (see Table 1 and Table 2). Live imaging, in vitro, and in vivo, is the most relevant approach to study the mechanisms underlying spine structure [16,18,21,24,25,26,28,38,44,45,70,94], however, to determine global changes in structural remodeling, the spine analysis after culture/tissue fixation enables to analyze larger number of spines. For in vitro imaging, primary neuronal cultures are mostly used, in which dendritic morphogenesis develops during first two weeks [95] and thereafter spines start to maturate. Therefore 19 to 23 days in vitro (DIV) is the most appropriate time point to perform structural analysis of mature dendritic spines [16,21,44]. To visualize dendritic spines, neuronal cultures can be transfected with plasmid encoding green or red fluorescent protein (GFP, RFP) under e.g., synapsin-1. However, when transfection with viral vector carrying shRNA or targeted gene overexpression is applied, then additional transfection using plasmid encoding fluorescent protein (GFP or RFP) is necessary to visualize cell morphology. The organotypic slice cultures are commonly transfected using biolistic method and stained with lipofilic carbocyanine (DiI) membrane dye that exhibits enhanced fluorescence upon insertion into the cell membrane [18,21]. The high photostability of the dye acts as an effective tool to visualize cellular architecture including dendritic spines [96]. Spines in acute brain slices after chemical fixation [97] are labeled with DiI [18,21,32,35,47,53,96] or Golgi staining [15,34,46,49,52]. The key element in dendritic spine staining is to apply an appropriate, gentle tissue fixation to assess the relevant effects on spine structure and eliminate cascades of biochemical processes affecting biophysical properties of neuronal membrane caused by cell death [32,96,98,99], therefore the vital condition of cells is an important factor in accurate spine analysis [97,100]. It is still not clear how in vitro, ex vivo, and in vivo models differ in speed of biochemical processes [97,100,101,102]. Therefore, the live cell imaging approach constitutes a suitable alternative, however, it requires analyzing more neurons, culture or animals per group due to technical limitations during live imaging. Imaging in vivo is usually performed in animals under anesthesia [38,94], sensory experience [27], or after behavioral training [29,45,47]. For in vivo live cell imaging, transgenic mice expressing fluorescent proteins (GFP/RFP/YFP) within whole brain, brain regions, or specific cell types are used [38,40,41,45,103]. It is worth emphasizing that several anesthetic drugs affect spine dynamics [104,105], which can constitute a crucial element during in vivo live cell imaging under anesthesia.

### 3.3. Microscopic Methods

In the recent years, the techniques enabling structural analysis of dendritic spines considerably evolved. The resolution of standard light microscopy (around 250 nm in a lateral plane) limits the precise detection of the fine details such as the spine neck, however, parameters such as length, head width, or area are still precisely detectable using standard confocal fluorescent light imaging [70]. Thus, fluorescence labeling of dendritic spines remains the most popular technique used to their visualization [1,62,66,67,71,72,81,85,106,107,108,109,110,111,112,113,114,115,116,117,118], see also Table 1 and Table 2. Accurate imaging of small details in dendritic spines structure, such as the spine neck, the thin filopodia or short spines became possible with the development of super resolution techniques [119,120,121]. The progress started with the development of stimulated emission depletion (STED) microscopy [122,123] followed by other techniques such as photoactivated localization microscopy (PALM) [124], stochastic optical reconstruction microscopy (STORM) [125,126] enabling imaging in nanoscale resolution. Investigation of all the parameters including neck width is achievable also using serial section electron microscopy (EM), which enables a detailed morphometric analysis at the nanoscale [127,128,129]. However, in the EM imaging, a sample needs to be fixated, permeabilized, dehydrated, and also placed under high vacuum—these procedures include the type of sample fixation (cryo- or chemical- fixation) that frequently causes structural artifacts to disturb the morphometric parameters of the dendritic spines [101,130,131] (comparative morphological analysis of images from live imaging and their respective images obtained using EM, see [130,131]). Currently, the most popular method used for precise dendritic spines imaging is based on super-resolution fluorescent light microscopy (lateral resolution around 20 to 40 nm in fixed tissue) through an expression of fluorescent membrane proteins that reduce an impact of sample preparation [101,132]. In STED microscopy, the use of continuous-wave lasers requires higher depletion beam power than with pulsed lasers, resulting in more severe photobleaching of the sample. Some of these constraints have been bypassed by the use of Switching Laser Mode (SLAM) microscopy, in which a switching between laser modes in the confocal microscope provides a way for diffraction-limited resolution images of spines and other structures. Although some laboratories have successfully used PALM and STORM to image live brain tissue and spines, their low imaging speed hinders the collection of high-resolution images in live samples [133,134,135,136,137,138,139,140,141]. Even though, the ultimate resolution achieved by stochastic microscopy (STORM and PALM) is comparable to electron microscopy, it happens when a very large number of emitter blinks is collected, otherwise the actual resolution is lower due to insufficient sampling density [142]. Thus, the imaging speed is the main factor limiting the application of stochastic microscopy techniques in live imaging and in high throughput analysis. Thus, the studies related to physiological and pathological processes of spine remodeling are mainly addressed to fluorescent confocal microscopy [16,18,19,21,22,29,42,43,44,46,47,50] due to the possibility to analyze the higher number of spines than in EM [23,33,36], and STED [27,37,38,39,40,41,45,48]. The mechanisms underlying spine structure are mainly performed using EM and STED due to the high-resolution imaging. Moreover, in STED imaging, small fields of view can be imaged rapidly, and when combined with 2P-excitation optical sectioning one can image at considerable depths (80–100 μm) in thick acute brain slices. Thus, STED allows imaging of live dendritic spines, providing a super-resolution view of the spine neck (length and diameter) and head, enabling an improved assessment of the spine structure-function relationship. Although the benefits of STED and PALM/STORM are evident, their current disadvantage is the need for high fluorescence labeling density in order to collect many photons per pixel to provide an acceptable signal-to-noise ratio [143]. However, the combined 3D-STED microscopy and fluorescent labeling of the extracellular fluid enables the development of super-resolution shadow imaging (SUSHI) [144], which greatly alleviates problems of photobleaching and phototoxicity associated with traditional imaging approaches [144]. SUSHI produces sharp negative images of all cellular structures, enabling unbiased imaging of unlabeled brain cells with respect to their anatomical context. Another improvement is achieved by the application of recently developed machine learning algorithms, that use high-resolution images to train neuronal networks and improve low-resolution and noisy datasets [145,146,147].

### 3.4. Image Analysis and Spines Morphology

**Figure 3:**
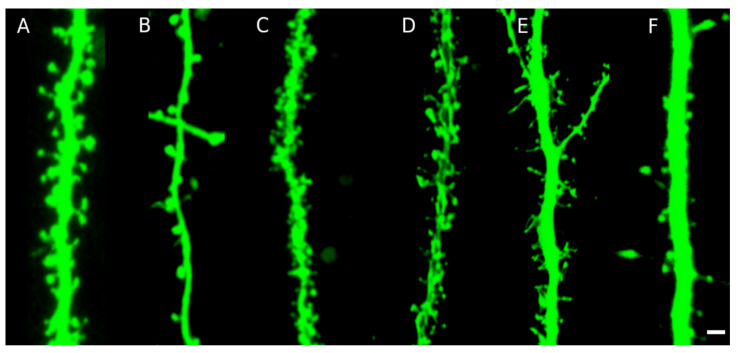
Different imaging modalities; fluorescent image of a dendrite with spines. Different types of neurons and staining techniques were used. The actual colors were changed in postprocessing to the green. (**A**) Live-cell imaging of primary hippocampal culture transfected with plasmid-encoded RFP (**B**) Confocal image combined with Airyscan processing of hippocampal fixed slices biolistically labeled with DiI staining (**C**) transcranial two-photon imaging of the motor cortex in a freely moving mouse. (**D**) Confocal image of organotypic hippocampal slice culture biolistically transfected with plasmid-encoded RFP. (**E**) Confocal image of fixed primary hippocampal culture transfected with plasmid-encoded GFP, (**F**) Confocal image of thick hippocampal brain slice marked with DiI. Scale bar: 2 μm.

The process of manual spine segmentation is very elaborate, thus several specialized automatic segmentation algorithms have been created, see [89,148] for a detailed review. Some of these methods utilize 2D maximal intensity projection of the image, due to insufficient resolution of traditional confocal microscopy in the axial direction, which does not allow to visualize sufficiently the detailed spine structure in 3D [149]. However, an accurate morphological quantification requires 3D reconstruction of the spine surface, which is still challenging both from the perspective of imaging and image segmentation. The software tools suitable for analyzing spine overall spine population [150] usually fail to accurately model individual spines 3D morphology, as discussed in [151]. The introduction of various types of pathological conditions, imaging of different brain regions, usage of different imaging methods and staining techniques, all result in images with different data modalities and with different artifact presence. An example of different data modalities is illustrated in Figure 3, where six different types of images are presented. We can immediately recognize the appearance of such artifacts as overlapping spines (Figure 3A,C,D), very thin spines at the limit of microscope resolution with detached fragments (Figure 3E,F), a halo around the dendrite (Figure 3A,C), the complicated and often branching structure of dendritic spines (Figure 3C,D), and inhomogeneity of spines and dendrite (Figure 3D). The diversity of images and a presence of artifacts is the major obstacle for using fully automatic segmentation algorithms, which often require setting the parameters controlling the segmentation according to data modalities and might be insufficiently flexible to cope with complicated structures [151]. Thus, many analyses are based on manual or semi-manual processing of images [152]. Several methods based on conventional/deep machine learning have recently been reported for automatic segmentation and analysis of dendritic spines [153,154,155]. However, the main obstruction to use machine learning algorithms in practical application is the absence of sufficient manually annotated data in 3D.

The most popular software tools that were reported in experimental protocols to be used for spines segmentation and analysis are SpineMagick (patent no. WO/2013/021001), 3dSpAn [152], Neurolucida [156], SpineLab [157], Imaris Editing Tools of FilamentTracer [158], NeuronIQ [159], MetaMorph [160], 3DMA-Neuron [161], NeuronStudio [162].

The dendritic spines are often classified as belonging to various morphological subpopulations such as stubby, mushroom, thin, filamentous, and filopodia—see Figure 2. Whether there are indeed certain distinctive clusters or we do observe a continuum of shapes, is still an open question [62]. Most categories were manually predefined, based on a visual inspection of the specified classification criteria. Recently, methods for unsupervised classification or non-classification approaches have been developed, see [163] for a review. Thus, to assess the morphometric changes, either a classification scheme can be followed, where the percentages in spines categories are compared [19,29,33,42,43,44,46], or alternatively, a direct comparison of certain morphological parameters is performed [16,18,19,21,23,29,34,36,37,42,47,48,49]. Quite often, a dimensionless ratio of certain parameters is analyzed, the advantage of such an approach is that it measures only the changes in spine geometrical shape independently of changes in size [16,18,19,21,29,47]. In the case of in vivo observations, paired tests, or analysis in changes of morphometric parameters is possible [16,21]. Spine generation or elimination is assessed by comparing the spines linear density (spines number per dendrite length) [22,36,37,38,39,40,41,42,43,44,45,47,49,50].

## 4. Sample Size, Data Analysis and Observed Effects

Choosing an appropriate sample size for an experiment aimed to measure quantitative effects is not a straightforward task. In practice, the sample size is mostly dictated by experimental capabilities rather than determined by a sort of statistical estimation. From a statistical point of view, the sample size shall be sufficiently large in order to guarantee that the false negative rate is adequately small, allowing for the successful detection of the sought effect, as well as reproducibility of results. The computer simulations [142] allow to connect the false negative rate with the underlying result magnitude, and the distribution of the morphological parameters. For example, we analyze the spine length, with eight animals per group and 60 spines per animal, assuming a 10% increase in spine length and an undetectability rate (false negative rate, type II error) of 40%. The difficulty is that even the rough a-priori assumptions of magnitude of potential changes is rarely legitimate and thus it is hard to justify estimated recommendations for optimal sample sizes. However, the simulation results might be used to estimate the reproducibility of the obtained results if we know the distribution of the morphological parameter and its changes. It has also been shown [142] that the diversity of dendritic spines, results in “heavy-tailed” distributions for some quantities, e.g., the spine length, where the effect is that changes in length are harder to detect than changes in head-width, whose distribution is more gaussian.

The minimal sample size used in animal models (behaviorally trained, genetically modified, or after intraperitoneal drug treatments) is determined to be five neurons per animal, with minimum of four animals, resulting in at least 200 spines per group up to more than 3000 spines per group [18,28,30,40,41,45,47]. However, in most cases, more animals were analyzed (usually six animals per group). To observe a significant effect in vitro, 8 to 10 cells per experimental condition from at least three independent cultures were analyzed, resulting in 24 to 30 neurons and 200 to 650 spines per experimental condition [16,18,21,29,44]. The spine density is typically determined by analyzing 1200 to 2000 µm of dendritic length per experimental group [15,47], or about 500 µm per dendritic shaft [38]. We favor analyzing at least 6000 µm of dendritic length per experimental group, from at least three different cultures, similarly for ex vivo as in vitro, for both cases, the spine density is comparable. Transcranial in vivo imaging involves a smaller number of analyzed spines, however, a minimum of six animals per group were analyzed [27,38,39,40,64,67]. Moreover, to reduce the possible differences in spine morphology and density caused by their location on dendrites within all of the mentioned models analyzed, spines should belong to secondary and tertiary distal dendrites [16,18,21,44,47].

The experiments exhibit the nested hierarchical structure groups-animals-cells-dendrites-dendrite branches-spines. Thus, the nested statistical tests [16], or ANOVA test with post-hoc tests (such as Tuckey’s test [15,17,18,19,25,30,31,32,43,44]; unpaired *t*-test with Welch’s correction [18,21,24,47], two-tailed Student’s *t*-test [22]; Mann-Whitney test [20,23,28,35,37], Fisher’s least square difference [38,42] are used when applicable. Other statistical variants include Dunn’s Test [46], linear regression with lines fitted to the density plots for each cell [27], simple linear regression and multivariate linear regression [34]; Kolmogorov-Smirnov test combined with Student’s t test or Mann–Whitney U test [26,29,36,40,41,50,51], correlation analysis using Pearson’s correlation [34,49].

Most of the observed effects involve changes in spines density, or creation/elimination rate for in vivo observations. Both the increase of spines density (6–135%) and the decrease (5–22%) have been reported, see the last column in Table 1 and Table 2. The parameters used for morphometric measurements include spine length, length/width ratio, head area, head volume, total spine volume, and neck diameter, see Figure 2B. The reported change of these parameters was in the range from 15% all the way up to 780% [49], the decrease of the morphometric parameters was also observed. These values are consistent with the simulation results, smaller changes remain mostly unnoticed and not significant statistically, for most of the reported sample sizes. Another quantification is based on categorizing spines into the classes discussed, and analyzing the changes in class populations [15,18,19,29,33,42,43,44,46] with reported changes in the range 2–150%.

## 5. Conclusions

In this review, we explored a quantitative analysis of dendritic spines within various biological models in physiological and pathological conditions. In particular, we pointed to key elements in each step of the methodological workflow, such as sample preparation, image acquisition, morphometric analysis, statistical approach, and selection of appropriate sample size. The selection of a stimulation or a disease model, and staining methods, obviously depends on the question underlying the experiment; studies investigating changes in individual spine morphology are usually addressed by live cell imaging, while studies related to global dendritic spine remodeling are usually performed in fixed samples.

The most popular imaging technique reported in summarized research, is fluorescent confocal imaging. It enables to visualize dendritic spines during live cell imaging, or directly in living organism by a transcranial window. However, due to the small spine size (1–5 µm), and the resolution limits (around 250 nm in the lateral plane), the fine details of the spine structure (such as neck width) could not be sufficiently quantified, thus the geometrical analysis and comparison of the spine shape is substantially limited. Accurate imaging is possible using an electron microscopy or by using super resolution techniques. These techniques, in turn, are complicated if applied in live cell imaging and they are mostly used with cells after chemical fixation. They also provide a much smaller number of spines, compared to confocal microscopy, at the same imaging time.

The adequate number of spines, cells, cultures, and animals, have to be analyzed according to applied statistics—these numbers vary within the testing conditions (in vitro/ex vivo/in vivo) and sample preparation (fixed vs. live sample). The statistical approach should include individual diversity within the cell type and animal, so that cell-nested or animal-nested statistics can be applied to observing the relevant effect. Size effect of spine remodeling and morphological changes, depends on the biological model used and has to be considered in relation to the applied experimental paradigm, and the type of stimulation. The key element in dendritic spine studies is to apply an accurate, quantitative morphometric spine analysis, which is still challenging both from the perspective of imaging and image segmentation. The diversity of images and the presence of artifacts is the major obstacle for using fully automatic segmentation algorithms, therefore many analyses are based on the manual or semi-manual processing of microscopic images. This is a time-consuming step, usually limiting the number of spines used in the quantitative analysis. The image segmentation algorithms perform much better with the high-resolution images obtained with super resolution techniques, in these cases the reconstructed spine number is limited by imaging capabilities, as already discussed. Thus, an accurate morphological spine quantification requiring 3D reconstruction is still challenging, both for available algorithms and imaging techniques, taking into account the requirement that the analysis is performed on thousands of spines per experimental condition.

Emphasizing the particular importance of each aforementioned methodological step in dendritic spines studies, it has to be taken into account that the structure-function relation may not be observed in experimental conditions, as might be expected. The reason may be caused by an insufficient number of spines, or an insufficient number of cells analyzed per experimental condition. The simulation results show that the spine diversity limits the detection of small morphological changes, thus many changes most likely remain undetected, as observing them requires analyzing a much larger spine population, which is usually practiced due to experimental limitations. Another reason behind the absence of observable changes might be a lack of activation of the signaling pathway regulating spine structure and function. It is well-known that the principal architectural component of the spine structure is the actin cytoskeleton [164], which underlies activity-dependent structural changes of spines [165,166,167]. The cycle of actin remodeling in spines is very dynamic (turn-over of actin monomers in filaments acts within every minute) and dependent on the dendritic spine compartment, e.g., a small population of actin in the base of the spine neck is more stable and remains in filaments for more than several minutes [167]. The balance between actin polymerization and depolymerization plays a critical role in structural plasticity of dendritic spines. Therefore, activation of various downstream signaling pathways may differentially affect the actin structure, leading to diverse functional modification, or even a lack of them [168]. Changes in the actin network are regulated by actin-binding proteins and their regulators influence many different aspects of actin dynamics, e.g., actin polymerization, depolymerization, trafficking, and various specific processes such as spine morphogenesis, maturation, motility and synaptic transmission (see Reviews [169,170,171]). Although the actin cytoskeleton is assumed to trigger spine formation, elimination, morphological and functional alteration, the mechanisms of actin reorganization by actin-binding proteins and their regulators, contributing to the function of spines and synapses, are still poorly understood. Signaling pathways that regulate structure and function of dendritic spines, are complex and their activity is transient and organized within specific spine compartments. Therefore, this area in neurobiological studies is still mysterious, despite increasing knowledge in the past years [170]. More detailed analysis within downstream signaling pathways may lead to better understanding of the mechanisms underlying structural synaptic plasticity. Interdisciplinary approaches, including super resolution imaging techniques, electrophysiology, and biochemical analysis, have to be implemented into further studies to characterize spatio-temporal changes within signaling pathways regulating spine structure.

## Figures and Tables

**Table 1:** The experiments assessing the morphology and remodeling of dendritic spines in physiology and under stimulation. The arrows indicate an increase ( ↑ ) or a decrease ( ↓ ) of measured parameters.

Biological Model and References	Sample Type Experimental Approach	Imaging Method	Analyzed Parameters	Sample Size	Software Used	Statistical Test Used	Quantitative Changes [%]
**Postsynaptic receptor antagonism** Treccani et al. 2019 *Mol Neurobiol* [15]	FSL and FRL rats Golgi Staining	Light microscope	Spine density, spine type density	6 neurons from 6 animal resulting in 36 neurons and 1200–2200 um analyzed dendritic length per group	Semi-automatic, ImageJ and Filament Tracer algorithm of the Imaris software	Two-way ANOVA with Tuckey’s post hoc test	75% ↑ in total density, 50–60% ↑ in specific spine type density (mushrom, long-thin)
**Postsynaptic receptor agonism** Bijata et al., 2017 *Cell Rep* [16]	Primary hippocampal culture, RFP-labeled neurons Organotypic hippocampal culture	Live cell imaging, fluorescent confocal microscopy,	length/head width ratio	8–25 neurons resulting in 326–631 per condition	SpineMagik software	Nested Anova	↑ in spine length, down in spine head
Murai et al. 2002 *Nat. Neurosci*. [17]	Hippocampal slices, DiI staining	Confocal microscope	Spine length, spine density	4 independent experiments, 1385 spines in total	ImageJ software	ANOVA	30% ↓ in spine length, 20% ↓ in spine density
**Genetic modification** Michaluk et al. 2011 *Sci. Rep.* [18]	Primary hippocampal culture, plasmid transfection carrying eGFP Organotypic hippocampal culture, eGFP biolistic plasmid transfection carrying eGFP Transgnic rats, DiI staining	Live cell imaging, Fluorescent confocal microscopy Live cell imaging, fluorescent confocal microscopy Confocal microscopy	Spine density, % of spine type (mushroom, thin, stubby, shuft) Length/width ratio	Min. 2 cells from each of 4 animals, resulting in 200 spines per group	ImageJ software	Unpaired Student’s *t* -test; one-way or two-way ANOVA and a post-hoc Tukey’s test.	2% ↓ in mushroom type, 3% ↑ in thin type; 52% ↑ Length/width
Lin et al. 2017 *J. Biol. Chem.* [19]	Primiary hippocampal culture, plasmid transfection carrying GFP	Fluorescent confocal microscopy	Spine type density (mushroom, thin, stubby), length, head width, neck width	3 independent experiments, 71–79 dendrites from 33 to 39 neurons in each group	MetaMorph software	Student’s t test or ANOVA followed by Tukey post hoc test	56% ↓ in mushroom type, 125–150% ↑ in thin and filopodium type
Bozdagi et al. 2010 *J. Neurosci.* [20]	Hippocampal slices, Nissl staining	Confocal microscopy	Spine volume, densisty	70 spines from 12 cells from 12 slices (cKOs), and 64 spines from 11 cells from 11 slices (control)	NeuronStudio	Mann–Whitney U test, Student’s t test, and ANOVA with Scheffe’s post hoc test	Transcient Increase in spine volume/enlagement, no effect on spine density
**Chemically induced** **LTP** Magnowska et al. 2016 *Sci. Rep.* [21]	Primary hippocampal culture Transgenic rats	Live Cell Imaging, fluorescent confocal microscopy GFP-labeled neurons Confocal Imaging, DiI staining	the length/width ratio, head width,	266–641 spines analyzed per group from 3 independent in vitro experiment	ImageJ and SpineMagik software	Unpaired Student’s *t*-test or an unpaired *t*-test with Welch’s correction or two—one-way ANOVA	↑ in head width (MMP-9 inhibition), ↓ in head width (TIMP-1 sequestration), spines are longer and thinner, in length/width ratio
Szepesi et al. 2013 *PLOS ONE* [22]	Primary hippocampal Cultures	Live cell imaging, fluorescent confocal microscopy, RFP-labeled neurons	Density of spine-head protrusion	5 cells per group	ImageJ software	Two-tailed Student’s *t*-test	365% ↑ in spine-head protrusion density
Borczyk et al. 2019 *Sci. Rep.* [23]	Organotypic hippocampal slice cultures	Electron microscopy	Spine volume spine density	4 slices resulting in 119 and 138 spines analyzed per group	Reconstruct sofware	Mann-Whitney test	30% ↑ in spine volume
Lang et al. 2004 *PNAS* [24]	Acute hippocampal slices from transgenic mice expressed EGFP	Two-photon microscope	Spines area	1155 spines from 20 slices	Custom-written software	Paired/unpaired *t*-test	Transcient expansion of spines ranging from 25–275%
Stein et al. 2021 *Cell Reports* [25]	Acute hippocampal slices from transgenic mice expressed GFP	Two-photon microscopy	Spine volume	1 segment of secondary or tertiary basal dendrite imaged for each neuron		Two-way ANOVA with Tukey’s multiple comparison test	Reduction of stimulaed spine volume
**LTD** Zhou et al. 2004 *Neuron* [26]	Acute hippocampal slices from neonatal rats	Two-photon microscope	Spine head diameter	18 neurons, 7–26 spines/neuron, total of 272 spines	ImageJ software	Student’s t test	>10% down in spine head diameter, in 75% of al analysed spines
**Sensory experience** Trachtenber et al. 2002 *Nature* [27]	Transgenic mice expressed GFP in V cortical layer of pyramidal neruons, Immunolabelling	Transcranial two-photon imaging, electron microscopy	Spine density		Custom image-acquisition MatLab software Neurolucida software	Linear regression lines were fitted to the density plots for each cell, Student’s two-tailed *t*-test.	
Majewska and Sur 2003 *PNAS* [28]	Mice expressing GFP in cortical layer 5 neurons, In vivo imaging	Two-photon microscope	Spine motility quantified as length change per time unit	4 mice, six cells, 149 spines	Custom written algorithms, manual analysis	Mann–Whitney U test	60% ↑ in spine motility at P28, 15% (up) at P42
**Spatial memory and learning** Bencisk et al. 2019 *Sci. Rep.* [29]	Primary hippocampal culture, EGFP-transfected Transgenic mice	Fluorescent confocal microscopy Electron microscopy	Spine type density (mushroom, filamentous, stubby), Head/neck width ratio, length	133–326 spines analyzed to determine spine type density per dendritic group from 3 independent cultures 552–561 from 3–5 mice per group	ImageJ software	Student’s *t*-test or non-parametric Mann-Whitney test or multiple-group comparisons Tukey post-hoc test	50% ↓ in mushrom spine type density
**Physical environmental stimuli** Kirov et al. 2004 *Neuroscience* [30]	Hippocampal slices of CA1 field, GFP expression	Two-photon microscopy,	Spine density	127–201 spines from 5–8 dendrites from 3–5 slices per animal, 5 animals per group	Imaris software, Huygens software	Two-way ANOVA, followed by Tukey’s post hoc test	↓ in spine density
Fiala et al. 2003 *J.Comp. Neurol*. [31]	Hippocampal slices, perfusion-fixed hippocampi	Electron microscopy	Spine density	56–86 spines reconstructed from serial sections at all time points	IGL Trace software	ANOVA. Tukey’s honest significance differences test	No difference
Trivino-Paredes et al. 2019 *J Neurophysiol.* [32]	Acute hippocampal slices, DiI staining	Confocal microscope	Spine density	2–3 dendritic segments for each cell, 15–34 slices	ImageJ software	One-way ANOVA test followed by a Tukey post hoc analysis	27% ↑ in males, 36% ↑ in females DG; 41% in females, 36% ↑ in males CA1

**Table 2:** The experiments assessing the morphology and remodeling of dendritic spines in brain diseases. The arrows indicate an increase ( ↑ ) or a decrease ( ↓ ) of measured parameters.

Biological Model and References	Sample Type Experimental Approach	Imaging Method	Analyzed Parameters	Sample Size	Software Used	Statistical Test Used	Quantitative Changes [%]
**Alzheimer disease** Androuin et al. 2018 *ActaNeuropathologica* [33]	Human biopsies, layers II–III of the right middle frontal gyrus, fixed Transgenic mice, fixed hippocampal slices	Electron microscopy	proportion of stubby and thin spines, neck diameter, volume, length,	3–5 patients per group, analyzed 22 spines in serial sections, counted in a 29 μm2 square at 50–100 μm from the pyramidal layer. A mean of 35 measurements was performed in one section (every two fields in two squares of the 400-mesh grid). 50 spines per mouse, resulting 200 spines/4 mice per group	ImageJ software	Two-way ANOVA test, to the fraction of spines with head and headless spines the arcsin function was applied for each animal, followed by two-way ANOVA.	30% ↑ in neck diameter, 35% ↑ in volume. 15% ↓ in length
Boros et al. 2019 *Neurobiol. Aging* [34]	Human postmortem brain tissue, Golgi-stained, fixed tissue, II and III cortical layers	Nikon Eclipse Ni upright microscope	Spine head diameter, length, density,	Min. 2 cells from each slice were analyzed resulting in 10–20 per group, density counted per 10 mm was determined for 10–20 dendrites and averaged.	Neurolucida 360, dendrites traced using semiautomated directional kernel algorithm	Simple linear regression, multivariate linear regression, Pearson’s coefficient and two-tailed unpaired *t*-tests.	
Smith et al. 2009 *PNAS* [35]	Acute hippocampal slices, fixed DiI staining, DiOlistic method	Confocal microscope	Spine density, area, length, head diameter	Min. 30 dendritic segments photographed for each condition	ImageJ software	Mann-Whitney U test	56% ↓ in spine density, 49% ↑ in spine area, 37% ↑ in head diameter, 22% ↑ in length
**Parkinson disease** Parajuli et al. 2020 *eNeuro* [36]	Mice, dorsolateral striatum, immunogold labeling, fixed slices	Electron microscopy (FIB/SEM imaging)	Spine density, head volume, neck length,	253–382 imaged section resulting in 7–10 dendrites and 109–177 spines per group (within whole study resulting in 65 dendrites and 1285 spines).	ImageJ software and Reconstruct Software, manual analysis	Distribution of spine head volume using Kolmogorov-Smirnov Z test; Student’s t test or Mann–Whitney U test; for more groups one-way ANOVA or Kruskal-Wallis test	65% ↓ in spine density, 180% ↑ in head volume
**Fragile X syndrome** Booker et al. 2019 *Nat. Comm.* [37]	Fmr1 KO male C57/Bl6J mice	2-photon, STED, SFB-SEM microscopy	Head length, Neck length, Head width, spine density,	6–11 dendrites were reconstructed from each mouse, which possessed a total of 38–49 spines (average = 4.4 spines/dendrite).	ImageJ software using deconvolved images	Mann–Whitney U-test	No effect
Nagaoka et al. 2016 *Sci. Rep.* [38]	Thy1-GFP mice	2-photon microscopy, Cranial window	Spine generation and elimination	7–15 intervals, 3–6 cells, 5–7 mice, 598 spines-3154 spines analyzed per group; The average length of the analyzed dendritic shafts was 488±60 µm from 126 cells	ImageJ Simple Neurite Tracer plugin	Fisher’s exact test, Kruskal–Wallis test, Steel’s test	80% ↓ in spine generation
**Down syndrome** Real et al. 2018 *Science* [39]	Human iPSC Transplanted to mice	2-photon longitudinal imaging	Spine density	monitored >500 dendritic segments from 6 mice per group	ImageJ software	Kruskal-Wallis test	
**Rett syndrome** Garre et al. 2020 *Nat. Comm.* [40]	Thy1-YFP transgenic mice, frontal association cortex, layer V	Transcranial 2-photon microscopy	Spine formation and elimination [%]	Min. 4 mice per group	ImageJ software	Unpaired two-tailed t or Mann–Whitney test, or paired two-tailed Wilcoxon tests	6% ↑ in spine elimination
Garre et al. 2017 *Nat. Med.* [41]	Thy1-YFP transgenic mice, frontal association cortex, layer V	Transcranial 2-photon microscopy	Spine formation and elimination [%]	Min. 4 mice per group	ImageJ software	Unpaired two-tailed t or Mann–Whitney test, or paired two-tailed Wilcoxon tests.	5% ↓ in spine density, 5–8% ↑ in spine elimination and formation
**Autism spectrum disorders** Gouder et al. 2019 *Sci. Rep.* [42]	Primary iPSC-derived pyramidal glutamatergic culture, GFP-labeled dendritic spines	Fluorescent confocal microscopy	Spine type, density, Spine mean diameter, volume, head volume, length	3–14 dendrites per group	Filament Tracer module of Imaris 7.6 software	Unpaired Student *t*-test, Fisher’s F-test.	
**Huntington disease** Puigdellívol et al. 2015 *Hum. Mol. Gen.* [43]	Mice, cortex, striatum	Confocal microscopy	Spine type (mushroom, thin), density	Spine density: 63–83 dendrites; n = 4 animals per genotype; spines counted in dendritic segments range from 15 to 40 μm of length. Spine type: cortex: 319 spines from 30 dendrites from 4 animals per genotype; striatum: 280 spines from 25 dendrites from 4 animals per group	ImageJ Plugin Cell Counter and “Polygon selections” tools to determine spine diameter	One-way ANOVA with Tukey post hoc comparisons	12% ↓ in density, 22% ↑ in mushroom spine type
**Schizophrenia** Lepeta et al. 2017 *EMBO Mol. Med.* [44]	Primary rat hippocampal neurons, plasmid transfection	Live cell fluorescent confocal imaging	Spine type (mushroom, thin), head area, spine density	6 cell from single experiment resulting in total 419–469 spines per group	ImageJ and SpineMagick Software, custom scripts written in Python with the NumPy, SciPy, and Matplotlib	Spine density was measured using two-way repeated-measures ANOVA with post hoc analysis by Tukey’s multiple comparisons.	15% ↓ in mushroom spine type, 5% ↑ in thin spines, 23% ↓ in head area
**Depression** Moda-Sava 2019 *Science* [45]	Thy1-YFP transgenic mice and C57BL/6J mice, prefrontal cortex	2-photon microscopy	Spine formation and elimination [%], spine density	Min. 50 dendritic segments (20–30 μm in length) per animal, 5–7 mice per group	ImageJ software	Spine density Kruskal-Wallis analysis of variance	8% ↑ in spine elimination, 14% in spine formation
Aguayo et al. 2018 *Front. Mol. Neurosci* [46]	Sprague-Dawley rats, Golgi Staining, fixed hippocampal tissue	Confocal microscopy	Spine type (stubby, mushroom, filopodia), density	5–7 of 80 µm dendritic segment in length, 6 cell per animal	-	Kruskal-Wallis test followed by Dunn’s Test	55% ↑ in thin spine type density
Krzystyniak et al. 2019 *Int. J. Mol. Sci.* [47]	Mice, DiI Staining	Confocal microscopy	Spine density, Length to head width ratio	5–17 cells per group, min. 6 animals per group	SpineMagik And 3dSpAn software	Unpaired Student’s *t*-test or nonparametric *t*-test with Welch correction	33% ↑ in spine density, 20% ↓ in length/head width
**Stroke** Wang et al. 2016 *PNAS* [48]	Mice, corticospinal neurons projecting to C8 spinal segments	2-photon microscopy	Spine density	20 neurons per group resulting in 800 dendritic segments (apical/distal: −200; apical/proximal: 300; and basilar: 300)	Dendrite reconstruction performed in Neurolucida and analyzed using NeuroExplorer	Multiple group comparisons were made using ANOVA, and post-hoc differences tested by Fisher’s probable least square difference	10% ↑ in spine density
**Epilepsy** Musto et al. 2016 *Sci. Rep.* [49]	Mice, Golgi staining,	Brightfield microscopy Axioplan 2 microscope	Spine length, Spine density	5–6 projection from 7 animals per group, min. 10 dendrites per animal for the brain structure.	ImageJ software	Correlation analysis using Pearson’s correlation	22% ↓ in spine density and length, 780% ↑ in length
**Prion disease** Fang et al. 2018 *PLOS Pathogenes* [50]	Primary hippocampal neurons stained with fluorescent phalloidin	Confocal microscopy	Spine density	Spine density analyzed from 15–24 cells from 3–4 independent experiments	ImageJ software	Student’s *t*-test	135% ↑ in spine density
**HIV infection** Alturi et al. 2013 *PLoS One* [51]	Neuroblastoma cells infected with clade B/C HIV-1 virus DiI staining	Confocal microscope	Spine density, Spine area, Spine length	20 optical serial sections of 0.14 µm/section per cell	ImageJ software	Student’s *t*-test	70–30% ↓ in spine density 90–60% ↓ in spine length depend on clade variant (B or C)
**Influenza infection** Hosseini et al. 2018 *J. Neurosci.* [52]	Neurons of CA1 and CA3 and dentate gyrus regions from mice infected with IAV Golgi staining	Vrightfield microscopy Axioplan 2 microscope	Spine density	4–5 animals, 10 cells per animal, 40–50 dendrites per group	ImageJ software	ANOVA	↓ in spine density CA1 17%, CA3 19% (H3N2 virus variant) CA1 22% CA3 15% (H7N7 variant)
**Toxoplasmosis** Parlog et al. 2014 *Dis. Models Mechanisms* [53]	Cortical and hippocampal neurons DiL staining, DiOlistic method	Brightfield microscopy Axioplan 2 microscope	Spine density, spine length, spine head width	5–23 dendrites, 3 independent experiments,	Neuroexplorer software	Two-tailed Student’s *t*-test	↓ in spine density, spine length, no effect on head width
**Antiviral responses** Chen et al. 2017 *EMBO Rep*. [54]	Cortical and hippocampal cultured neurons GFP transfected Brain section of somtosensory cortex from transgenic mice, YFP signal	Fluorescence microscope	Spine density, spine head width,spine length	3 dendrites from each cell resulting in 45–48 dendrites from 15–16 neurons per group obtained from 3 independent experiments 43–50 cells from 3 mice per group	ImageJ software	Unpaired *t*-test	↑ in spine density, ↓ in spine head width
